# Microwave ablation of 105 T1 renal tumors: technique efficacy with a mean follow-up of two years

**DOI:** 10.1177/0284185120956283

**Published:** 2020-09-10

**Authors:** Vanessa Acosta Ruiz, Pär Dahlman, Einar Brekkan, Maria Lönnemark, Anders Magnusson

**Affiliations:** 1 Department of Surgical Sciences – Radiology, Uppsala University, Uppsala Sweden; 2 Department of Surgical Sciences – Urology, Uppsala University, Uppsala Sweden

**Keywords:** Ablation procedures, kidney, percutaneous, computed tomography

## Abstract

**Background:**

Thermal ablation (TA) with radiofrequency (RFA) or cryoablation (CA) are established treatments for small renal masses (≤4 cm). Microwave ablation (MWA) has several potential benefits (decreased ablation time, less susceptibility to heat-sink, higher lesion temperatures than RFA) but is still considered experimental considering the available small-sample studies with short follow-up.

**Purpose:**

To evaluate technique efficacy and complications of our initial experience of renal tumors treated using percutaneous MWA with a curative intent.

**Material and Methods:**

A total of 105 renal tumors (in 93 patients) were treated between April 2014 and August 2017. MWA was performed percutaneously with computed tomography (CT) guidance under conscious sedation (n=82) or full anesthesia. Patients were followed with contrast-enhanced CT scans at six months and yearly thereafter for a minimum of five years. The mean follow-up time was 2.1 years. The percentage of tumors completely ablated in a single session (primary efficacy rate) and those successfully treated after repeat ablation (secondary efficacy rate) were recorded. Patient and tumor characteristics as well as complications were collected retrospectively.

**Results:**

The median patient age was 70 years and median tumor size was 25 mm. Primary efficacy rate was 96.2% (101/105 tumors). After including two residual tumors for a second ablation session, secondary efficacy was 97.1% (102/105). Periprocedural complications were found in 5.2% (5/95) sessions: four Clavien-Dindo I and one Clavien-Dindo IIIa. One postprocedural Clavien-Dindo II complication was found.

**Conclusion:**

MWA has high efficacy rates and few complications compared to other TA methods at a mean follow-up of two years.

## Introduction

Minimally invasive image-guided thermal ablation (TA) is an established treatment option for renal cell carcinoma (RCC) (1,2). Increasing evidence (1,3–9) suggests comparable oncological outcomes for partial nephrectomy (PN), radiofrequency ablation (RFA), and cryoablation (CA) in the treatment of T1a renal tumors. Advantages of TA include fewer and less severe peri- and postprocedural complications, greater preservation of renal function, and a shorter hospital stay compared to surgical excision (3,10). Percutaneous treatment under conscious sedation allows treatment of patients who pose an unacceptable anesthetic risk (11). Ablation is restricted by tumor size and location, with lower technique efficacy for tumors that are centrally located and >3 cm (11–13). Therefore, TA should be reserved for tumors where complete ablation can be achieved (1). Microwave ablation (MWA) is a heat-based ablative technique associated with decreased ablation and procedural times and requiring less sedation than RFA and CA (14). MWA is suggested to achieve higher intra-lesion temperatures, larger ablation zones, and to be less susceptible to heat-sink effect than RFA (14,15). Although MWA is an emerging method, there are limited long-term follow-up data (1,2).

Initial experience of percutaneous MWA-treated renal tumors for patients with a minimum follow-up interval of 12 months is reported. The aim of the present study was to evaluate technique efficacy and complications of renal tumors treated percutaneously using MWA with a curative intent.

## Material and Methods

### Patient recruitment

The Uppsala regional ethical review board granted approval for the study (Dnr 2012/518). The initial treatment decision was made by a team of subspecialty radiologists and urologists with six years of previous experience of renal tumor treatment with minimally invasive nephron-sparing procedures (220 percutaneous renal RFA and 77 laparoscopic or robot-assisted partial nephrectomies). Patient selection was based on the CT imaging findings and histopathology if available. Patients selected for MWA treatment were those with renal tumors, T1a, solitary kidney, impaired renal function, bilateral tumors, and/or predisposition for developing multiple tumors. Patients with T1b tumors were considered if severe co-morbidities and/or unacceptable surgical risks were present.

Between May 2014 and August 2017, 156 patients with 173 localized tumors (1–4 tumors per patient) were treated with MWA for a renal tumor. After informed written and verbal consent, these patients were assessed retrospectively in September 2018. Inclusion criteria were as follows: primary ≤T1b renal tumors with diagnostic biopsies (RCC and oncocytomas) with MWA as the primary treatment method, with a curative intent, for the untreated renal tumor. Follow-up needed to include contrast-enhanced CT (CE-CT) scans with a minimum follow-up of ≥1 year.

Patients with non-diagnostic biopsies (n = 28) or benign tumors other than oncocytomas (n = 12) were excluded to minimize any selection bias if these “intention-to-treat” tumors were to be included. Including RCCs only (and oncocytomas, as 25% of these have a chance of being an RCC (16)) was considered to give a more accurate reflection of MWA efficacy rates. Further exclusion criteria were: patients treated for metastases in the kidney (n = 2); patients in whom MWA was not the primary treatment method (n = 6); patients who could not undergo follow-up with CE-CT scans (n = 6; due to contrast medium allergy, n = 1); patients with previous underlying chronic kidney disease (n = 4) or reduced renal function after ablation (n = 1); and patients with follow-up <1 year (n = 9). The remaining 93 patients included had a total of 105 tumors (85 patients with single tumors, five patients with two tumors, two patients with three tumors, and one patient with four tumors).

Patient data (age and gender) and tumor characteristics (tumor size, modified RENAL nephrometry score [m-RNS] (17), histopathology) at the time of treatment were collected.

#### MWA procedure and follow-up

Preprocedural planning with CE-CT was the day before treatment. Baseline CT images were acquired in unenhanced and contrast-enhanced phases (corticomedullary, nephrographic, and excretory phases). A contrast medium was used (Iomeron, Iomeprol, 400 mL I/mL, Bracco imaging SpA, Milano, Italy) with an injection volume of 1 mL/kg (maximum of 80 mL) and flow rate of 4 mL/s. Preprocedural imaging evaluation and MWA treatment were performed by three radiologists (PD, ML, AM) with 15–35 years of experience in CT-guided interventions.

Ablation sessions were either under conscious sedation or full anesthesia, depending on patient co-morbidities, or patient and operator preference. In the case of a patient with multiple tumors, all tumors were treated during the same session. Treatments were under percutaneous CT guidance (Somatom Definition Flash, Siemens, Forchheim, Germany). Hydrodissection was used to displace organs >2 cm away from the intended ablative margin to avoid ablation of non-targeted structures. For this purpose, 100–500 mL of 50 mg/mL glucose (Glucos, Fresenius Kabi AB, Uppsala, Sweden) mixed with contrast agent (20 mL per L Omnipaque 300 mg I/mL, GE Healthcare Limited, Little Chalfont, UK) were percutaneously infused between the renal tumor and the adjacent structure. For tumors adjacent to the pelvo-ureteric junction, a ureter catheter and pyelo-perfusion was used to protect the pelvo-ureteric junction. After positioning the MWA antenna within the renal tumor, 1–3 core biopsies were taken. MWA was with the Emprint™ ablation system and thermosphere technology antennas (Medtronic, Minneapolis, MN, USA). A single antenna was used, using 100 W, and the ablation duration varied depending on tumor size. The manufacturers’ recommendations were followed to achieve an ablation margin of ≥5 mm. The antenna could be repositioned and further ablation performed if the first ablation zone did not cover the entire index tumor.

Immediate post-ablation imaging (with the same four-phase protocol as above) was used to assess ablation margins and procedural complications. In the case of residual tumor on immediate assessment, the antenna was repositioned and additional ablations were performed, with no additional contrast-enhanced control. Patients were admitted for observation and discharged within 24 h if no complications were observed.

Follow-up time was from the day of treatment to the last performed follow-up CT during the time of the study. Follow-up imaging included CT scans (with the same four-phase protocol as above) at six months and annually after treatment. Patients with local tumor progression were readmitted for retreatment. The mean follow-up time was 2.1 years with a maximum follow-up of 4.6 years.

### Image analysis and definitions of success

**Fig. 1.**
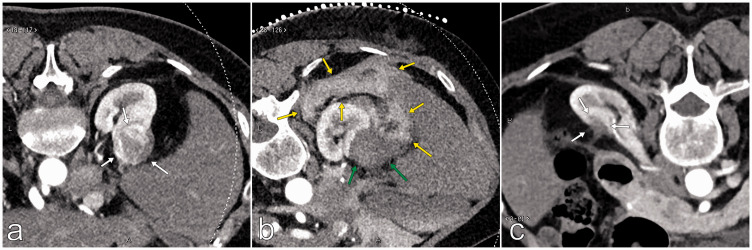
Example of a completely ablated renal tumor (clear-cell carcinoma) with MWA. (a) Preprocedural CE-CT showed a 33-mm tumor, m-RNS 10a (white arrows). (b) Hydrodissection was used to increase the distance between the liver and renal tumor (yellow arrows). After a total ablation time of 12 min, immediate CE-CT control showed a completely ablated tumor (green arrow). (c) At the six-month follow-up, CE-CT showed complete tumor treatment, without contrast enhancement in the treated area (white arrows). CE-CT, contrast-enhanced computed tomography; m-RNS, modified RENAL nephrometry score; MWA, microwave ablation.

**Fig. 2.**
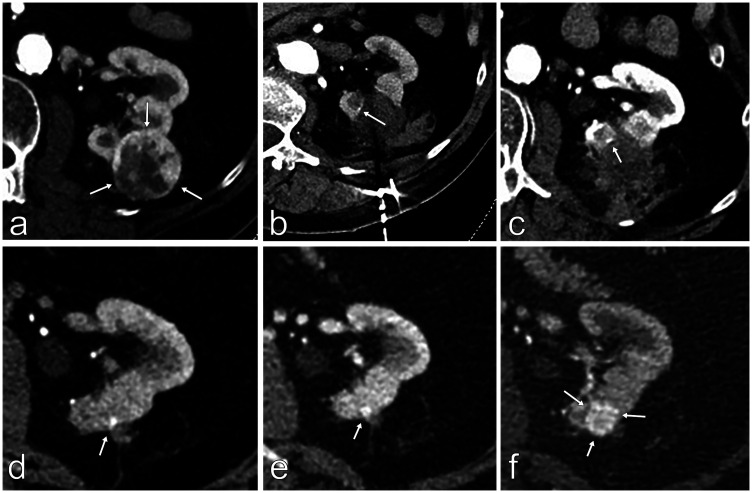
Example of a tumor treated with MWA. (a) Pretreatment CT showed a 3.5-cm tumor, m-RNS 9p points (arrows). (b) After treatment with 100 W (ablation time 10 min), a small part at the tumor border (arrow) was not fully treated. The antenna was repositioned, for further ablation (a further 3 min). (c) At the six-month follow-up, there was a high suspicion of residual tumor at the ablation margin (arrow). (d–g) The patient was followed at 1 year (d), 1.5 years (f), and 2 years (g) after ablation, verifying the successively growing residual tumor (arrow). This patient is currently planned for a second ablation session. CT, computed tomography; m-RNS, modified RENAL nephrometry score; MWA, microwave ablation.

Preprocedural and follow-up CT images were retrospectively reviewed (from the date of the procedure to December 2018) by a senior uro-radiologist together with a resident in radiology. The results were reported with the approved terminology (18) of the Society of Interventional Radiology (SIR). Complete tumor ablation was achieved if there was contrast enhancement < 20 HU at the index tumor site and ablation margins (Fig. 1). Residual tumor was defined as ≥20 HU in any part of the treated tumor/margin (Fig. 2). Local tumor progression was found when a contrast enhancement of ≥20 HU was seen at the ablated site after at least one follow-up study showing no contrast enhancement.

Efficacy rates were based on the comparison of baseline CT images of the index tumor with all follow-up CT images. The primary efficacy rate was the percentage of tumors resulting in complete ablation results (after a single session) on the CT scan at the six-month follow-up. The secondary efficacy rate was the percentage of tumors completely ablated after all sessions performed during the time of the study. Complications (events leading to morbidity and/or disability that might increase the level of care) during treatment or postprocedural admission were recorded and classified according to the Clavien-Dindo classification (19). Side effects were undesired consequences not leading to substantial morbidity.

## Results

Patient and tumor characteristics are presented in Table 1. Of the 105 tumors (in 93 patients), 103 tumors (in 91 patients) were treated in a single session and two tumors treated with a second ablation session. Of the total 95 ablation sessions, 82 were under conscious sedation and 13 under full anesthesia. Hydrodissection was used in 58 sessions and a ureter catheter was used in six patients. Median ablation time was 5 min/tumor (range = 2.5–20 min) and 1–5 antenna positions were applied (one position, n = 61; two positions, n = 32; three positions, n = 9; four positions, n = 4; and five positions, n = 1).

**Table 1.** Patient and tumor characteristics.

**Patient characteristics (n = 93)**	
Age (years)	70 (34–87)
Gender distribution (men / women)	58 / 35
**Tumor characteristics (n = 105)**	
Tumor size (mm)	25 (10 – 42)
T1a	101
T1b	4
Tumor side distribution (right / left)	50 / 55
m-RNS score (points)	7 (4–11)
*Tumor complexity (m-RNS points)*	
Low (4–6)	44 (42)
Medium (7–9)	45 (43)
High (10–12)	16 (15)
*Histopathology*	
Clear cell	53
Papillary type 1	22
Papillary type 2	2
Chromophobe	9
Oncocytoma	19

Values are given as n, n (%), or median (range).

**Fig. 3.**
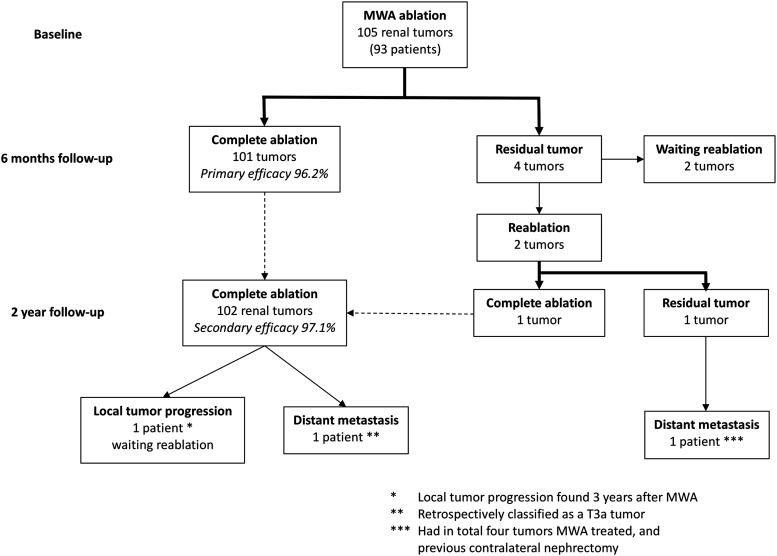
Flow chart showing results after MWA renal tumor treatment. Primary efficacy rate: percentage of tumors resulting in complete ablation results after a single session. Secondary efficacy rate: percentage of tumors completely ablated after all sessions performed during the time of the study. Thick line indicates result after MWA sessions. Dotted line indicates tumors summed to calculate the secondary efficacy rate. MWA, microwave ablation.

Primary efficacy rate was 96.2% (101/105 tumors). All of the four tumors not completely ablated in a single session were clear-cell RCC (ccRCC). Two residual tumors were planned for a second ablation session (Fig. 2), resulting in complete ablation for one of the tumors. Secondary efficacy rate was 97.1% (102/105) (Fig. 3).

One patient showed local tumor progression two years after a single MWA session. One patient treated in a single session for a 4.2 cm, centrally located tumor (m-RNS 10ah) had no residual tumor after ablation but progressed with pulmonary and adrenal metastasis three years after ablation. The biopsy-verified pulmonary metastasis was retrospectively correlated to unspecific nodular findings <4 mm in the pulmonary parenchyma found before ablation. In addition, the renal tumor grew further into the renal hilum than initially suspected, demonstrating renal vein invasion. This tumor, reassessed as a T3a tumor (previously categorized as T1b), should have been suitable for another primary treatment method other than ablation. This patient died three years after ablation and palliative management of metastasized ccRCC. During follow-up, four more patients died of causes unrelated to the treated renal tumor (Fig. 3).

The tumor resulting in a residual tumor after a second session also progressed with skeletal and lymph node metastasis, ending in palliative management. This 2.6-cm, central, endophytic tumor (m-RNS 8p) was found in a patient treated with MWA for four tumors in total. The patient had undergone contralateral total nephrectomy five years previously due to a 6.5-cm renal tumor with a similar appearance to the ones later ablated (Fig. 3).

Periprocedural complications were found in 5/95 (5.2%) sessions: four classified as Clavien-Dindo I (three cases of hematomas related to the placement of the MWA antenna, one case of a small pneumothorax) and one as Clavien IIIa. The Clavien IIIa complication was a pneumothorax occurring after subcutaneous infiltration of local anesthesia before ablation of a tumor in the upper pole. The pneumothorax was immediately treated with chest drain. One postprocedural complication was found (Clavien-Dindo II): a retroperitoneal hematoma (7.5 × 11 cm) requiring blood transfusion and vasopressor treatment. Three patients suffered side effects: two patients experienced nausea, low-grade fever and vomiting 24 h after the procedure and one patient experiencing pain during ablation.

## Discussion

In the present study, MWA had a high efficacy rate after renal tumor treatment on an intention-to-cure basis, with a low rate of complications.

The efficacy rates (primary = 96.2%, secondary = 97.1%) were comparable to those in a meta-analysis (20) reporting a primary efficacy rate of 97.6% for 616 malignant renal tumors. The development of a MWA technique has contributed to high rates of efficacy. An early study demonstrates recurrence rates up to 38% (21) and non-uniform ablations with low power generators. Antennas developed later (as used in this study), with both an active antenna cooling (reducing conductive heating) and high-power generators, contribute to more effective ablation zones (15). In addition, the larger (≤2 cm) active heating zone leads to higher intra-tumoral temperatures (15,22) making MWA less affected by tumor size (23–25). In comparison, RFA primarily depends on passive tissue heating and efficacy is reduced with increasing tumor size >3 cm (11,24). The size limit at which MWA can be expected to result in complete tumor ablation is still not defined. Although MWA of tumors >4 cm have resulted in primary efficacy rates of 87.5%–90% (26,27), current reports are limited by small sample sizes. When excluding the T1b tumor, which was retrospectively recategorized as a T3a tumor, all our T1b tumors (n = 3) were completely ablated in a single session. Further studies will need to assess the size limit at which complete ablation can be expected with newer MWA technology.

Local tumor progression after MWA is reported to be in the range of 0%–17% (20). The inclusion of only centrally located tumors could explain the relatively high (17%) local tumor progression rate in one study (28). Tumors adjacent to the renal pelvis are found to increase the potential of local tumor progression occurrence (25). However, MWA is reported to be less affected by the heat sink effect than RFA (29), which could explain why local tumor progression rates after MWA are low (around 2.1% (20)). Similar to this study, Klapperich et al. (23) found only one case of local tumor progression (25 months after ablation) after treating 100 T1a renal tumors. The slow growing nature of these, often indolent, small renal masses could account for the low occurrence of local tumor progression and why it appears long after treatment (30).

Few studies found distant metastasis; Chan et al. (31) found only two cases of distant metastases after treating 80 tumors. Another study of 58 patients report two deaths at 11 and 19 months after metastatic progression of the disease (32). Two patients were found to have distant metastasis in the present study, one of them showed an aggressive renal cancer disease already prior MWA (with previous history of multiple renal tumors and contralateral nephrectomy due to a renal tumor) and the other patient was initially underestimated as a T1b but retrospectively recategorized as a T3a tumor. The short follow-up time (≤2 years) in these studies limits further evaluation of the risk of distant metastasis after treatment. Nevertheless, mid-term MWA results after T1a tumor treatment are promising, revealing a similar trend to previous RFA and CA studies (3,4,33,34). Long-term studies will evaluate whether MWA can rival RFA and CA, which now present equivalent results with partial nephrectomy for ≥T1b tumors and a reported five-year recurrence-free survival rate of 90%–95.2% (1,3,4,8,34).

The four tumors that were not completely ablated after a single session were all ccRCC. Similarly, Shakeri et al. (35) reported that three of their four tumors resulting in local tumor progression were ccRCC. Theoretically the highly vascularized ccRCC could contribute to some variability in how the microwaves propagate (29,36). The effect of MWA on different RCCs has yet to be evaluated. In the present study, oncocytomas were included for MWA treatment, limiting comparison with other studies that do not include oncocytomas (20,23). At our institution, oncocytomas are considered for treatment as these tumors have been identified as RCC after renal mass biopsy in one of four cases (16).

There were few complications in the study (5.6%, 6/107 sessions), only one being a major complication (Clavien IIIa). A meta-analysis found an incidence of 17.5% for minor complications and 1.8% for major complications after MWA (20). The slightly higher rate of major complications after RFA (4.7%) and CA (7.5%) (37) could be explained by the multiple renal capsule punctures when placing multiple electrodes/needles needed to acquire larger (>3 cm) ablation zones (14). In contrast, MWA can be performed using a single antenna (15). Unfortunately, only immediate postprocedural complications were collected due to the wide geographical spread of the patients causing difficulties identifying complications 24 h after discharge. Late MWA complications can occur; a study (23) reports a late complication rate of 6% (6/100), all being asymptomatic urinomas (without significant decline in renal function) detected at a mean of 173.5 days after ablation. Still, complication rates for TA therapies are few, and are reported less frequently than for surgical management of small renal masses (3,10).

This single-center retrospective study has several limitations. A five-year follow-up would have been preferable to enable comparison with results of other TA techniques and surgical alternatives (33); however, a shorter follow-up is unavoidable when evaluating a new ablative modality. Analyses of factors affecting efficacy rates (e.g. tumor size, tumor complexity, and patient characteristics) were not evaluated; however, this was not the primary aim. Further renal tumor classification (e.g. Fuhrman or ISUP grading) would have been desirable to assess, but these were not always reported. Impact on renal function after treatment was not evaluated, although it seems not to be negatively affected (14). As a single MWA device was used, the results may not be comparable with other systems, although reduced ablation and procedural times and dosage of sedative medication favors MWA rather than RFA and CA (14).

In conclusion, the MWA results after a mean follow up of two years present a similar trend to early reports of established TA techniques. With comparable efficacy rates and low incidence of complications, MWA can potentially be included as a TA alternative but will have to be further assessed in studies with a longer follow-up.
